# Clinical verification of vimentin/EpCAM immunolipid magnetic sorting system in monitoring CTCs in arterial and venous blood of advanced tumor

**DOI:** 10.1186/s12951-021-00929-x

**Published:** 2021-06-16

**Authors:** Yan Liu, Qiuying Li, Tingsong Chen, Tianhao Shen, Xufeng Zhang, Ping Song, Lantao Liu, Jianming Liu, Tinghui Jiang, Xiaofei Liang

**Affiliations:** 1 Department of Interventional Oncology, Putuo Hospital, Shanghai University of Traditional Chinese Medicine, No. 164, Lanxi Road, Shanghai, 200333 China; 2 Second department of oncology, The Seventh People’s Hospital Affiliated to Shanghai University of Chinese Medicine, Shanghai, China; 3 Department of Thoracic Surgery, Putuo Hospital, Shanghai University of Traditional Chinese Medicine, Shanghai, China; 4 Huzhou Lieyuan Medical Laboratory Co., Ltd, No. 800, Rujiadian Road, Huzhou, 313009 China

**Keywords:** Advanced tumor, Circulating tumor cells, Arterial blood, Venous blood, Gene mutation

## Abstract

**Background:**

Circulating tumor cells (CTCs) are the dominant factor leading to tumor metastasis. This study aims to investigate the effect of disparate sources of CTCs on the treatment and prognosis of patients with advanced tumors by analyzing the number and gene mutations change of CTCs in arterial and venous blood in patients with advanced tumors.

**Results:**

A CTCs sorting system was constructed based on Vimentin-immunolipid magnetic balls (Vi-IMB) and EpCAM immunolipid magnetic balls (Ep-IMB). Results showed that the prepared Ep-IMB and Vi-IMB had lower cytotoxicity, better specificity and sensitivity. The number of arterial CTCs was higher than that of venous CTCs, with a statistically significant difference (P < 0.05). Moreover, the prognosis of the low positive group of total CTCs in arterial blood and venous blood was higher than that of the high positive group, with a statistical significance (P < 0.05). The genetic testing results showed that the targeted drug gene mutations in tissues, arterial CTCs and venous CTCs showed a complementary trend, indicating that there was heterogeneity among different tumor samples.

**Conclusions:**

CTCs in blood can be efficiently captured by the CTCs sorting system based on Vi-LMB/Ep-LMB, and CTCs detection in arterial blood can be utilized to more accurately evaluate the prognosis and predict postoperative progress. It is further confirmed that tumor samples from disparate sources are heterogeneous, providing a reference basis for gene mutation detection before clinical targeted drug treatment, and the detection of CTCs in arterial blood has more potential clinical application value.

*Trial registration*: The Ethics Committee of Putuo Hospital, PTEC-A-2019-18-1. Registered 24 September 2019.

**Graphic abstract:**

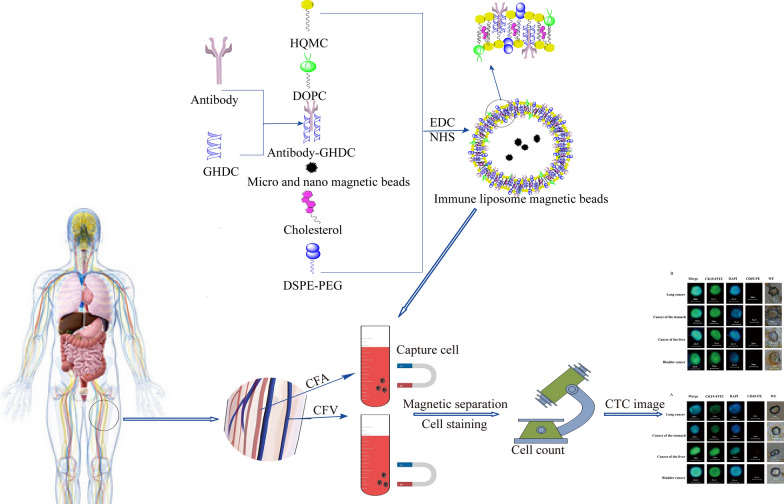

**Supplementary Information:**

The online version contains supplementary material available at 10.1186/s12951-021-00929-x.

## Background

Cancer is a risk factor that seriously affects the health of residents, the burden it caused has continued to increase worldwide in the past decade. Tumor deaths account for 1/4 of all causes of death in China, ranking the first, increasing by about 4% every year, and an annual increase of about 2% after adjusting for age factors [1, 2]. Tumor recurrence and metastasis are still the leading causes of death in patients with cancer [3, 4]. In the traditional view, the formation of cancer metastases is considered to be the terminal event of tumor occurrence and development. However, recent studies have demonstrated that even in the early stage of cancer, tumor cells can enter the blood circulation system directly or indirectly via the lymphatic pathway, and then colonize in distant tissues or organs and eventually proliferate to form metastasis. The part of tumor cells that are detached from the primary tumor or metastatic tumor and enter the blood circulation is called circulating tumor cell (CTCs). The appearance of CTCs is the premise and basis for distant metastasis [5, 6]. Consequently, the detection of CTCs in peripheral blood of patients with tumor is of great clinical significance in early diagnosis, disease progression and prognosis judgment, and real-time monitoring of curative effects of breast cancer, colorectal cancer, lung cancer, gastric cancer, liver cancer, pancreatic cancer, etc. [7–12].

It has been found that the formation of CTCs is related to epithelial mesenchymal transition (EMT). Under the regulation of EMT related transcription factors Snai, Twist, Zeb and E47 [13, 14], the expression of epithelial cell specific proteins, such as epithelial cell adhesion molecule (EpCAM), E-cadherin and cytokeratin [15, 16], were down regulated in tumor cells located in the original site, while mesenchymal specific proteins such as N-cadherin and vimentin were overexpressed. Tumor cells undergo EMT to acquire the characteristics of mesenchymal cells, showing stronger migration and invasion capabilities. They then evolve into tumor cells in the systemic circulatory system by crossing the surrounding stroma, invading the blood vessels and entering venous and arterial blood [17, 18]. Only a very small percentage of CTCs that enter the bloodstream survive a variety of challenges. Mesenchyma-epithelial transition (MET) occurs after the surviving CTCs reach the destination to form metastatic foci. The half-life of CTCs in the blood is as short as only 1–2.4 h, while the blood circulation of the human body is once a few seconds on average. Therefore, CTCs can be planted on disparate organs to form tumor metastasis after multiple blood circulation [19].

Prior to this study, a study on the spatial heterogeneity of CTCs was carried out by Yun-Fan Sun et al. [19] via animal model, in which the propagation route and characteristics of CTCs in patients with localized tumor were mapped, and CTCs was proved to have obvious heterogeneity in number, size and clustering state, indicating that the activation of EMT program in CTCs mainly occurs in the process of migration. It has been reported by a variety of studies on the relationship between tumor cell metastasis and epithelial-mesenchymal transition. CTCs changes the proliferation of cell phenotypes from primary tumors via the process of epithelial-mesenchymal transformation (EMT), which enables cells to play an important role in penetrating blood vessels for metastasis [20, 21]. It has also been further shown in some studies that EMT exists in various malignant tumors of epithelial origin, including lung cancer, colorectal cancer, breast cancer and pancreatic cancer, etc. [22, 23], suggesting that EMT is a process of epithelial transformation to mesenchymal phenotype, during which it will lead to the expression of Vimentin and loss of epithelial markers (EpCAM). Recent approaches to CTCs analysis based on epithelial cell adhesion molecule (EpCAM) have shown limitations in detecting CTCs in patients with tumors [24]: some CTCs express only epithelial or mesenchymal markers. Studies have shown that vimentin is highly expressed in various tumor cells, especially in tumor cells with EMT [25]. Consequently, vimentin has become a potential target for capturing CTCs in patients with tumor. In view of this fact, an accurate and efficient CTCs analysis method based on EpCAM+/Vimentin+ is developed in this study.

This research is the first time to study the relationship between the prognosis of patients by detecting the number of CTCs in arterial blood and venous blood, and gene detection was also used for the first time to compare the gene mutation in clinical samples of tissue, arterial CTCs and venous CTCs. This study was carried out to provide a technical reference for efficacy evaluation, prognosis judgment and detection of micrometastases in patients with cancer, and a reference basis for selection of clinical samples for gene mutation detection prior to clinical targeted drug therapy.

## Results

### Testing process

**Fig. 1:**
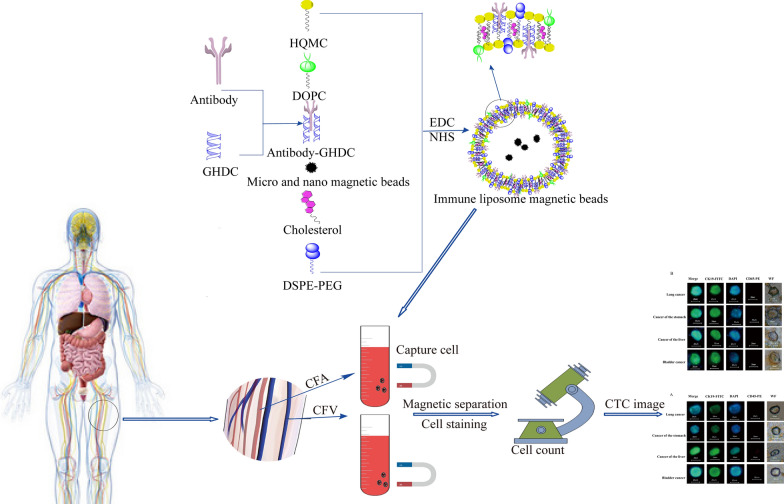
Schematic diagram of preparation of magnetic balls and separation and identification of CTCs in blood

In this study, immunolipid magnetic balls were prepared and coupled with antibodies to capture and separate CTCs from arterial blood and venous blood of tumor patients. The captured CTCs were identified and counted by a fluorescence microscope. The flow chart of preparation of magnetic balls and separation and identification of CTCs in blood is shown in Fig. 1.

### Characterization test results

**Fig. 2:**
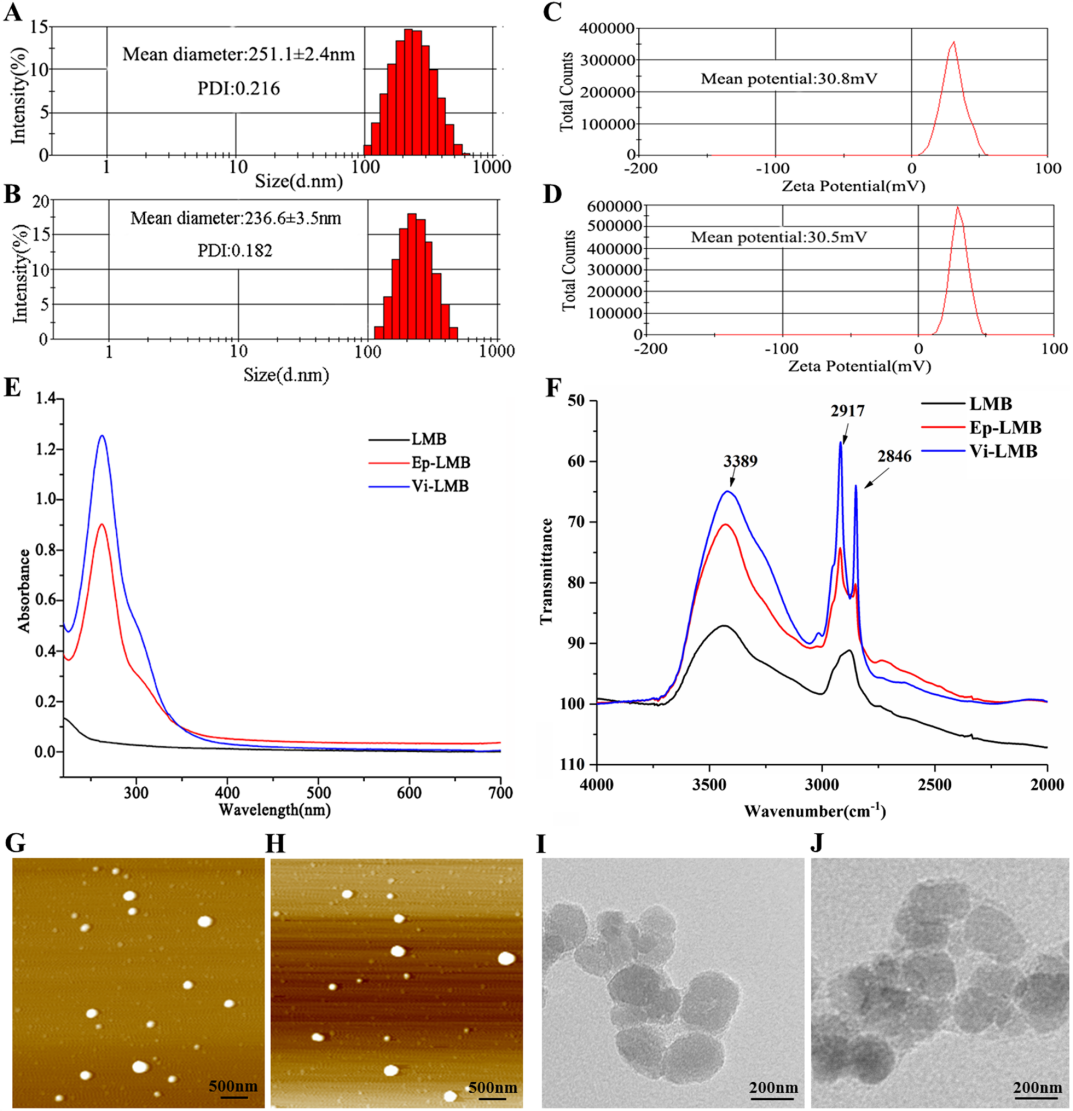
Characterization test of the magnetic ball. **A** Ep-LMB particle size distribution diagram; **B** Vi-LMB particle size distribution diagram; **C** Ep-LMB potential distribution diagram; **D** Vi-LMB potential distribution diagram; **E** UV test results of Ep-LMB and Vi-LMB; **F** Infrared test results of Ep-LMB and Vi-LMB; **G** Atomic force test results of Ep-LMB; **H** Atomic force test results of Vi-LMB; **I** TEM observation results of Ep-LMB; **J** TEM observation results of Vi-LMB

The characterization test results of Ep-LMB and Vi-LMB were shown in Fig. 2. The average particle size of Ep-LMB was 251.1 ± 2.4 nm, with a polydispersity index (PDI) of 0.216 (Fig. 2A). The average particle size of Vi-LMB was 236.6 ± 3.5 nm, with a PDI of 0.182 (Fig. 2B). The particle size test results showed that Ep-LMB and Vi-LMB are very small in particle size. The size of the particles in the solution determines the stability of the solution, indicating that the magnetic balls prepared in this study are characterized by superior stability. The potential test results showed that the charge of Ep-LMB was + 30.8 mV (Fig. 2C), and that of Vi-LMB was + 30.5 mV (Fig. 2D), the two kinds of magnetic balls were positively charged. The surface charged microspheres boasted favorable dispersibility due to electrostatic repulsion between each other, which was conducive to the dispersion of microspheres in hydrophilic solution. Moreover, positively charged liposome magnetic beads can easily bind to negatively charged cells; Fig. 2E showed the UV test results of Ep-LMB and Vi-LMB. Ep-LMB and Vi-LMB have absorption peaks at 260–280 nm, showing the characteristics of protein ultraviolet absorption. The FT-IR spectrum was shown in Fig. 2F. In the FT-IR spectra of Ep-LMB and Vi-LMB, new peaks were found at about 2840–2930 cm^−1^ due to the long carbon chain and methyl groups on the quaternary ammonium salt, indicating the presence of GHDC on both Ep-LMB and Vi-LMB. GHDC was conjugated with EpCAM and Vimentin respectively, indirectly indicating that EpCAM and vimentin antibodies have been conjugated on the surface of Ep-LMB and Vi-LMB. The results of atomic force microscope observations (Fig. 2G, H) showed that Ep-LMB and Vi-LMB were spherical in different sizes with regular shapes and no agglomeration, which indicated preferable stability and vesicle characteristics of liposomes. As can be seen from the observation results of transmission electron microscopy (Fig. 2I, J), Ep-LMB and Vi-LMB were circles of different sizes, with a diameter distribution between 200 and 300 nm and a relatively uniform distribution.

### Cytotoxicity

The MTT assay was used to determine the cytotoxicity of Ep-LMB and Vi-LMB binding cells (Additional file 1: Figure S1). Ep-LMB and Vi-LMB immunomagnetic beads had little effect on the activity of different cancer cells, and there was no difference in cell activity with the increase of immunomagnetic bead concentration. The IC50 of the two magnetic beads on various cancer cells was greater than 10 M without affecting cell viability. As a result, the interaction between Ep-LMB and Vi-LMB immunomagnetic beads and cells indicated that the immunomagnetic beads had low cytotoxicity and would not affect the capture and identification of CTCs in the later stage.

### Distribution of magnetic balls on the cell surface

**Fig. 3:**
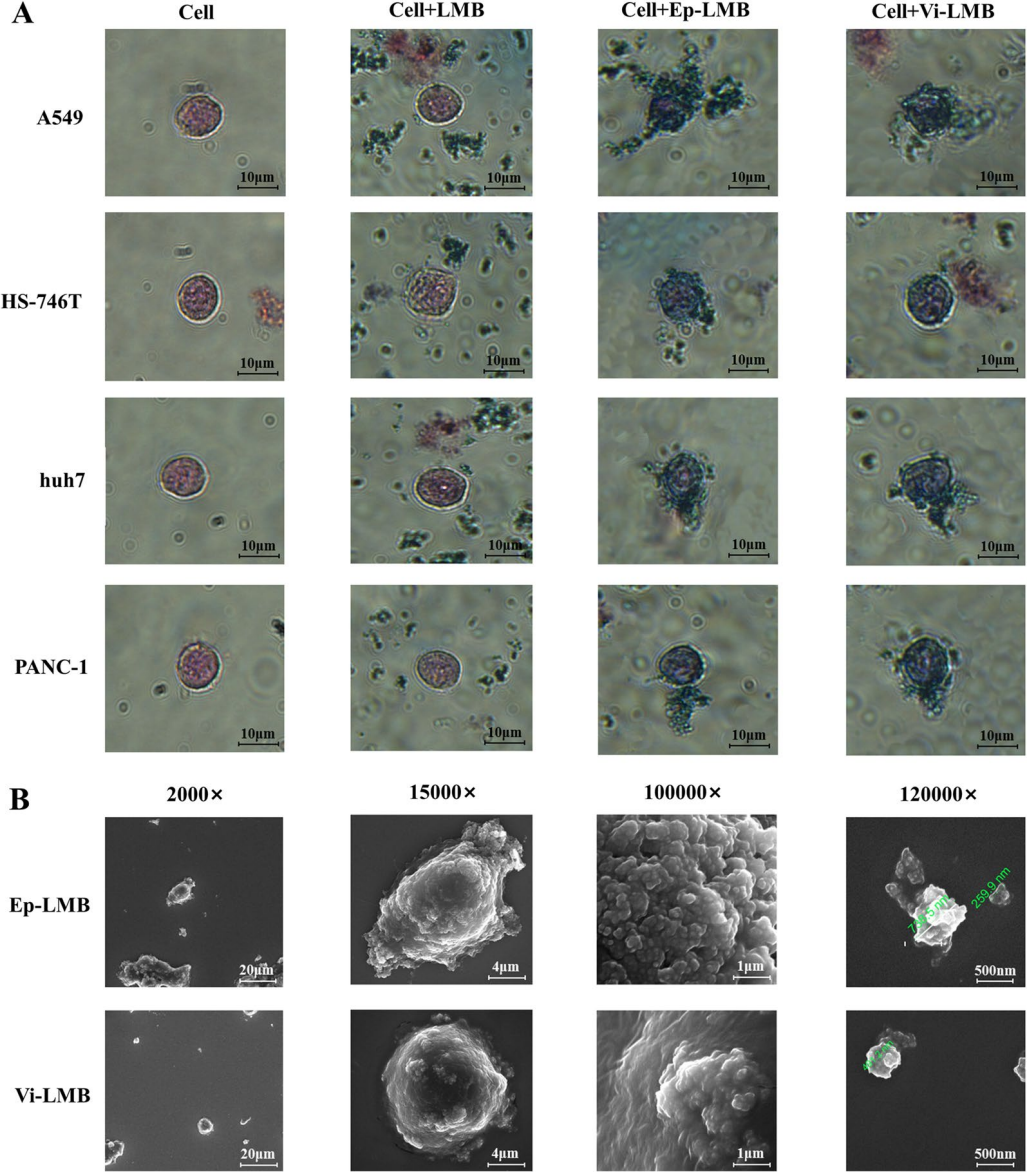
Prussian staining and SEM observation of the distribution of magnetic balls on the cell surface. **A** Prussian staining results after magnetic ball targeting and identifying cells; **B** SEM observation results after magnetic ball targeting and identifying cells

The results of Prussian staining (Fig. 3A) showed that the four kinds of cells were in good growth state, presenting a typical regular cellular morphology. LMB had no ability to recognize cells and distributed irregularly on and around the cell surface. Both Ep-LMB and Vi-LMB can target and recognize tumor cells, and they were mainly attached to the cell surface, indicating that Ep-LMB and Vi-LMB have target recognition capabilities. The observation results of SEM showed that the size of Ep-LMB and Vi-LMB was much smaller than that of the cells (Fig. 3B). It can be seen that the magnetic balls around the cells were enriched on the cell surface under a field of view of 2000×, and only a few magnetic balls were freely distributed; It can be seen under the field of view of 15,000× and 100,000× that a large number of magnetic balls were adsorbed on the cell surface, and the magnetic balls produced by the aggregation of these magnetic balls were enough to capture the cells; It can be clearly seen under the field of view of 120,000× that the magnetic balls were clearly distributed between 200 and 300, which was consistent with the results of magnetic ball characterization test.

### Binding time of magnetic ball and cell

**Fig. 4:**
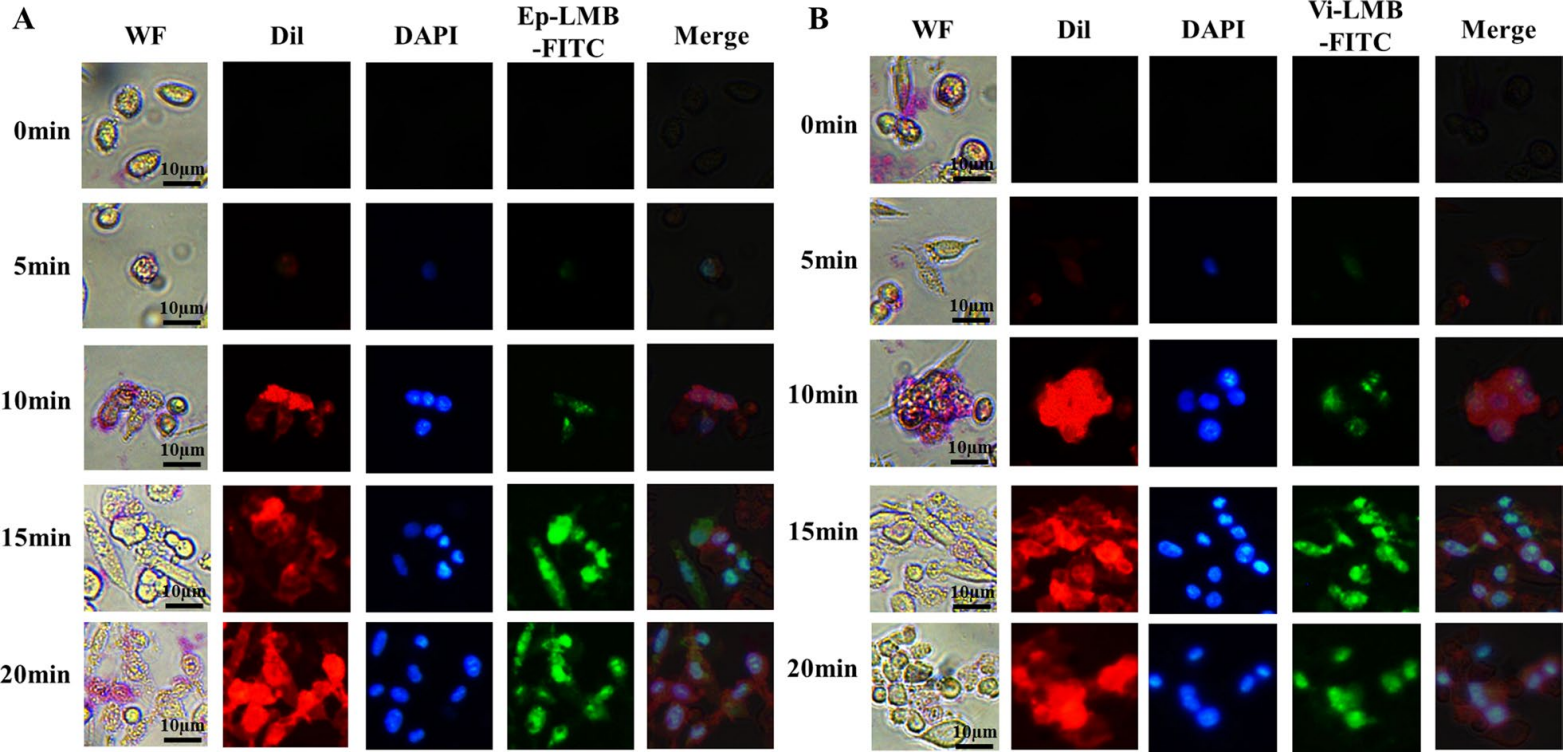
Binding time exploration of immune lipid magnetic balls and cells. **A** Changes in the binding of Ep-LMB-FITC and A549 cells at different time; **B** Changes in the binding of Vi-LMB-FITC and A549 cells at different time

In order to explore the best time of interaction between cells and immunolipid magnetic ball, Ep-LMB and Vi-LMB were labeled with FITC fluorescence respectively and added into the culture dish. At the same time, the cell membrane probe Dil and nuclear fluorescent dye DAPI were added. After staining, the cells were observed under a fluorescence microscope. The results in Fig. 4 showed that the FITC fluorescence signal in the cells increased gradually with the prolongation of time, indicating that the lipid magnetic balls on the cell surface increased gradually with the prolongation of time, and the best effect could be achieved when the incubation time was 15 min.

### Test results of cell capture efficiency

The capture efficiency of cells is shown in Additional file 2: Figure S2. Under the conditions of the same cell concentration and the same amount of magnetic beads, the capture rates of the five capture schemes showed an increasing trend (Additional file 2: Figure S2A), but 20 μL Ep-LMB/Vi-LMB sequential capture was considered the best capture scheme, with a capture rate of more than 90%. The verification was performed on simulated blood (Additional file 2: Figure S2B), and it was concluded that the capture efficiency would be reduced due to the viscosity of blood in the blood simulation system. In addition, the capture rate of A549, HS-746 T, huh7 and PANC-1 cells was detected to evaluate the stability of the combined efficiency of Ep-LMB/Vi-LMB and sequential capture. The detection results on PBS were shown in Additional file 2: Figure S2C. The combination of Ep-LMB/Vi-LMB and sequential capture had no significant difference in the capture rate of the four cancer cells (P > 0.05), with an average capture rate of over 90%. The detection results in simulated blood were shown in Additional file 2: Figure S2D. The combination of Ep-LMB/Vi-LMB and sequential capture had no significant difference in the capture rate of the four cancer cells (P > 0.05), with an average capture rate of over 85%. The results of cell gradient experiments with different antibody content showed that the capture efficiency reached the maximum when the antibody content was 60 μg (Additional file 2: Figure S2E).

### Cell immunofluorescence identification

**Fig. 5:**
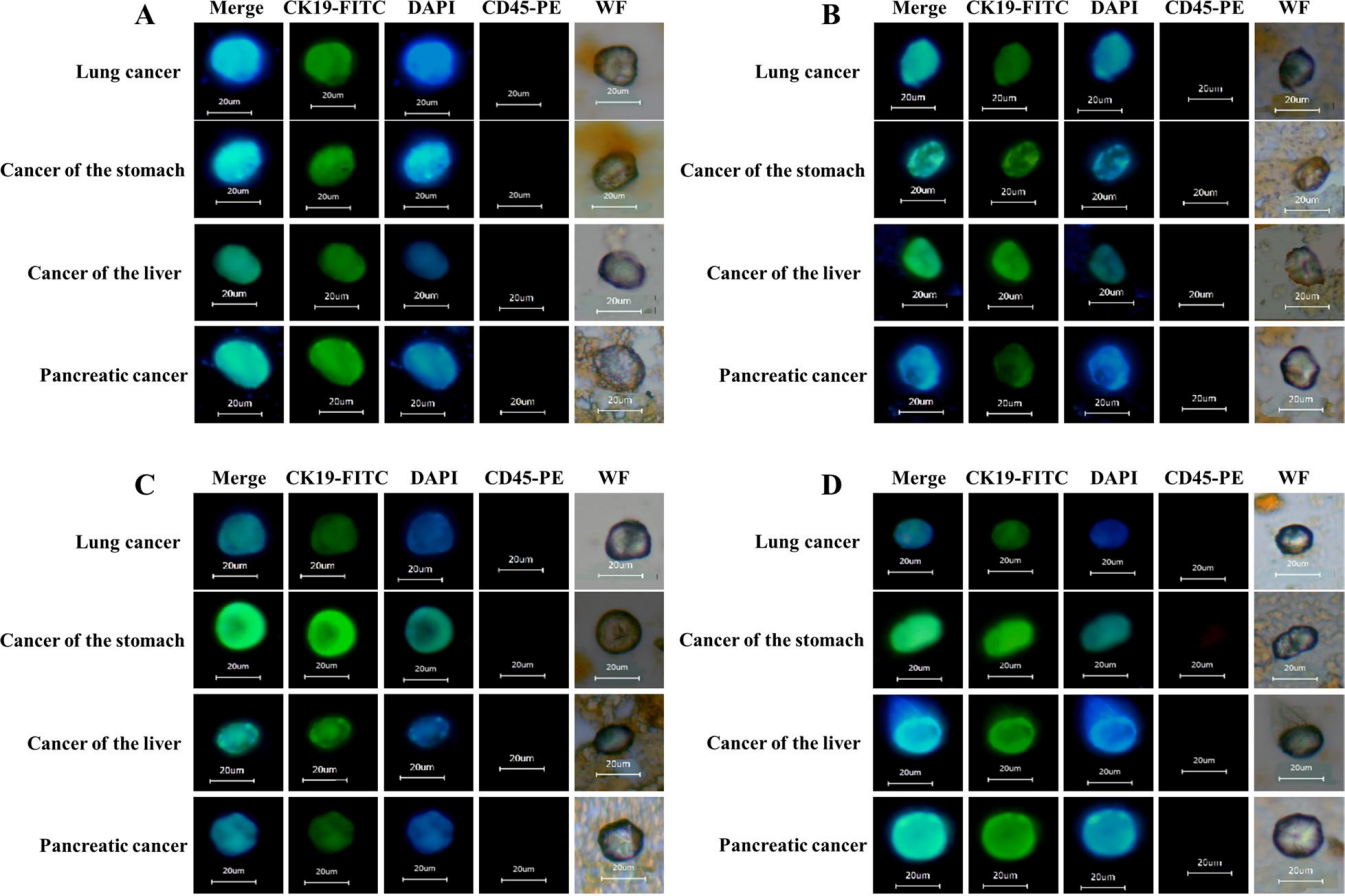
Immunofluorescence identification of CTCs in the blood of patients with tumor. **A** Ep-LMB captures CTCs in arterial blood of patients with different tumors; **B** Vi-LMB captures CTCs in arterial blood of patients with different tumors; **C** Ep-LMB captures CTCs in venous blood of patients with different tumors; **D** Vi-LMB captures CTCs in venous blood of patients with different tumors

The separated CTCs were subjected to smear observation, as shown in Fig. 5, in which there was obvious cell morphology under white light, CK19-FITC that identifies the tumor cells was positive, showing green fluorescence, DAPI that identifies the nucleus was positive, showing blue fluorescence, and CD45 used to exclude white blood cells was negative, that is, tumor cells did not show red fluorescence. Cells that meet the above-mentioned fluorescence coloring conditions can be judged as CTCs.

### Distribution of CTCs in the blood of patients with cancer

**Fig. 6:**
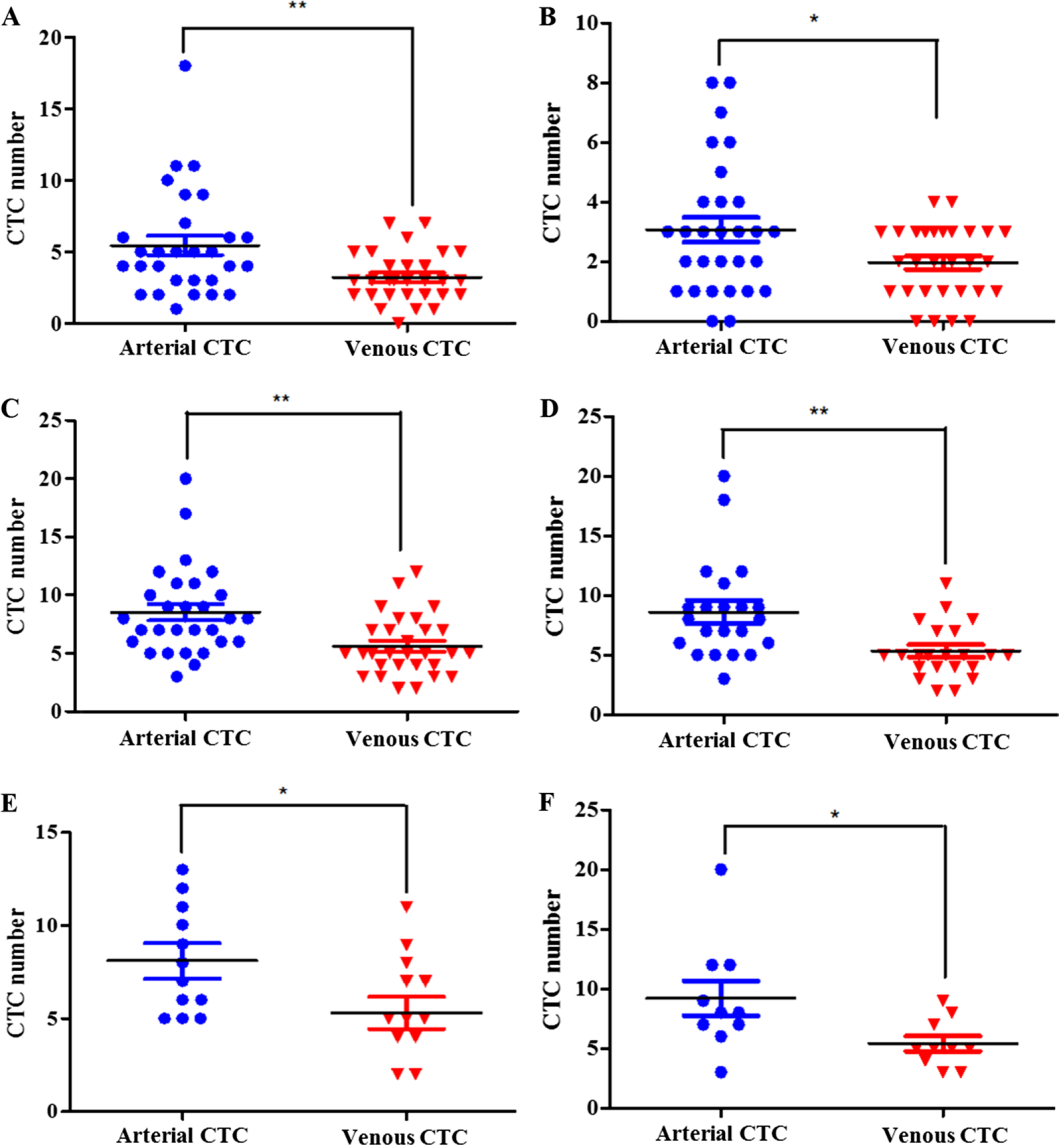
Distribution of CTCs in arterial and venous blood. **A** Captured by Ep-LMB; **B** Captured by Vi-LMB; **C** Captured by Ep-LMB combined with Vi-LMB; **D** Captured by Ep-LMB combined with Vi-LMB in case of tumor metastasis; **E** Captured by Ep-LMB combined with Vi-LMB in case of single tumor metastasis; **F** Captured by Ep-LMB combined with Vi-LMB in case of multiple tumor metastasis

As can be seen from the distribution of CTCs in arterial and venous blood captured by Ep-LMB, the amount of CTCs captured by Ep-LMB in arterial blood was higher than that in venous blood, with a statistically significant difference (*p* < 0.05) (Fig. 6A). As can be seen from the distribution of CTCs in arterial and venous blood captured by Vi-LMB, the amount of CTCs captured by Vi-LMB in arterial blood was higher than that in venous blood, with a statistically significant difference (*p* < 0.05) (Fig. 6B). As can be seen from the distribution of total CTCs in arterial and venous blood captured by Ep-LMB combined with Vi-LMB, the number of CTCs in arterial blood was higher than that in venous blood, with a statistically significant difference (*p* < 0.01) (Fig. 6C). Among cases with metastasis, the total number of CTCs in arterial blood was detected to be greater than that in venous blood, with a statistically significant differences (*p* < 0.01) (Fig. 6D); The total number of CTCs in arterial blood was greater than that in venous blood in case of single metastasis, with a statistically significant difference (*p* < 0.05) (Fig. 6E); The total number of CTCs in arterial blood was greater than that in venous blood in case of multiple metastases, with a statistically significant difference (*p* < 0.05) (Fig. 6F).

### Analysis of clinical correlation

The correlation between the total number of CTCs and clinicopathology of patients with cancer was analyzed, and the results are shown in Table 1. No significant correlation was found between gender, age and metastatic status of patients and the number of CTCs (P > 0.05).

**Table 1:** Correlation analysis of CTCs in peripheral blood of patients with cancer and clinical

	n	Arterial blood CTC			Venous blood CTC		
		Epithelial	Interstitial	Total CTCs	Epithelial	Interstitial	Total CTCs
		P value	P value	P value	P value	P value	P value
Gender							
Male	29	0.356	0.228	0.459	0.257	0.149	0.386
Female	39						
Age							
≥ 70	45	0.462	0.313	0.229	0.586	0.233	0.382
< 70	23						
Metastasis type							
No metastasis	46	0.216	0.328	0.247	0.263	0.281	0.132
Single metastasis	12						
Multiple metastasis	10						

### Survival analysis

**Fig. 7:**
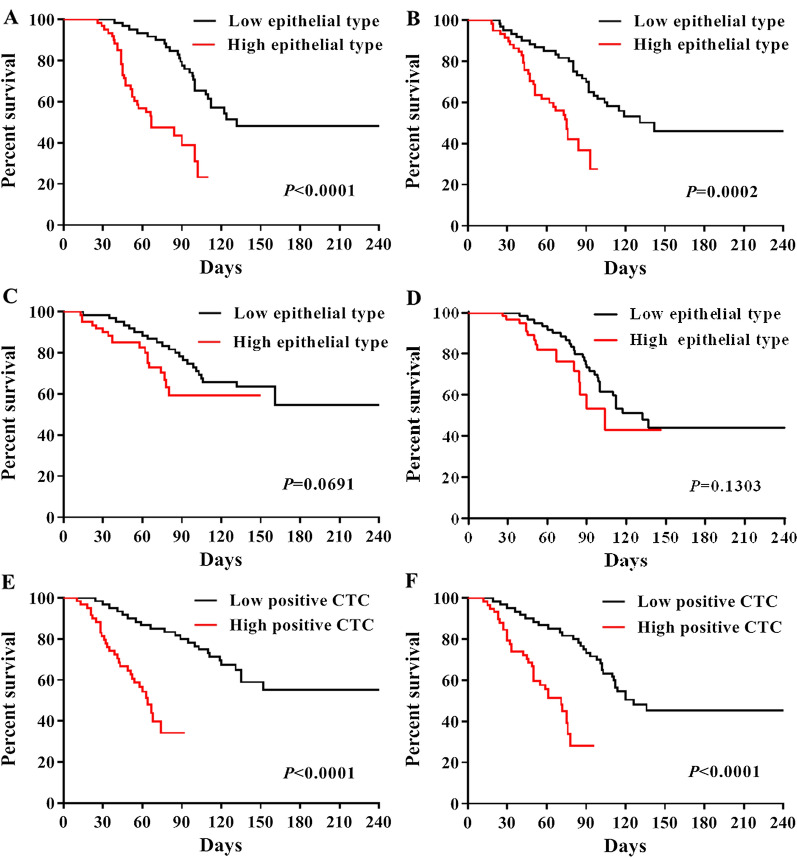
Survival curve of patients with cancer. **A** Correlation between the number of CTCs in arterial blood captured by Ep-LMB and the survival time of patients; **B** Correlation between the number of CTCs in venous blood captured by Ep-LMB and the survival time of patients; **C** Correlation between the number of CTCs in arterial blood captured by Vi-LMB and the survival time of patients; **D** Correlation between the number of CTCs in venous blood captured by Vi-LMB and the survival time of patients; **E** Correlation between the total number of CTCs in arterial blood and the survival time of patients; **F** Correlation between the total number of CTCs in venous blood and the survival time of patients

In this study, 68 positive cases were enrolled. As of the follow-up time, 50 cases had progressed, 18 cases had no progress, with a median progression-free survival of 3.9 months. For the purpose of exploring the impact of the total number and type of CTCs on the prognosis of patients, patients were divided into groups based on the average number of the total number, the proportion of epithelial type and the proportion of interstitial type, and the survival analysis was performed with the occurrence of progression as the endpoint event (Fig. 7). The prognosis of the low epithelial group of CTCs in arterial blood and venous blood was better than that of the high epithelial group, with a statistically significant difference (Fig. 7A, B); The prognosis of the low interstitial group and the high interstitial group of CTCs in arterial blood and venous blood had no statistically significant difference (Fig. 7C, D); The prognosis of the low total positive group of CTCs in arterial blood and venous blood was better than that of the high positive group, with a statistically significant difference (Fig. 7E, F).

### Gene detection assay

**Fig. 8:**
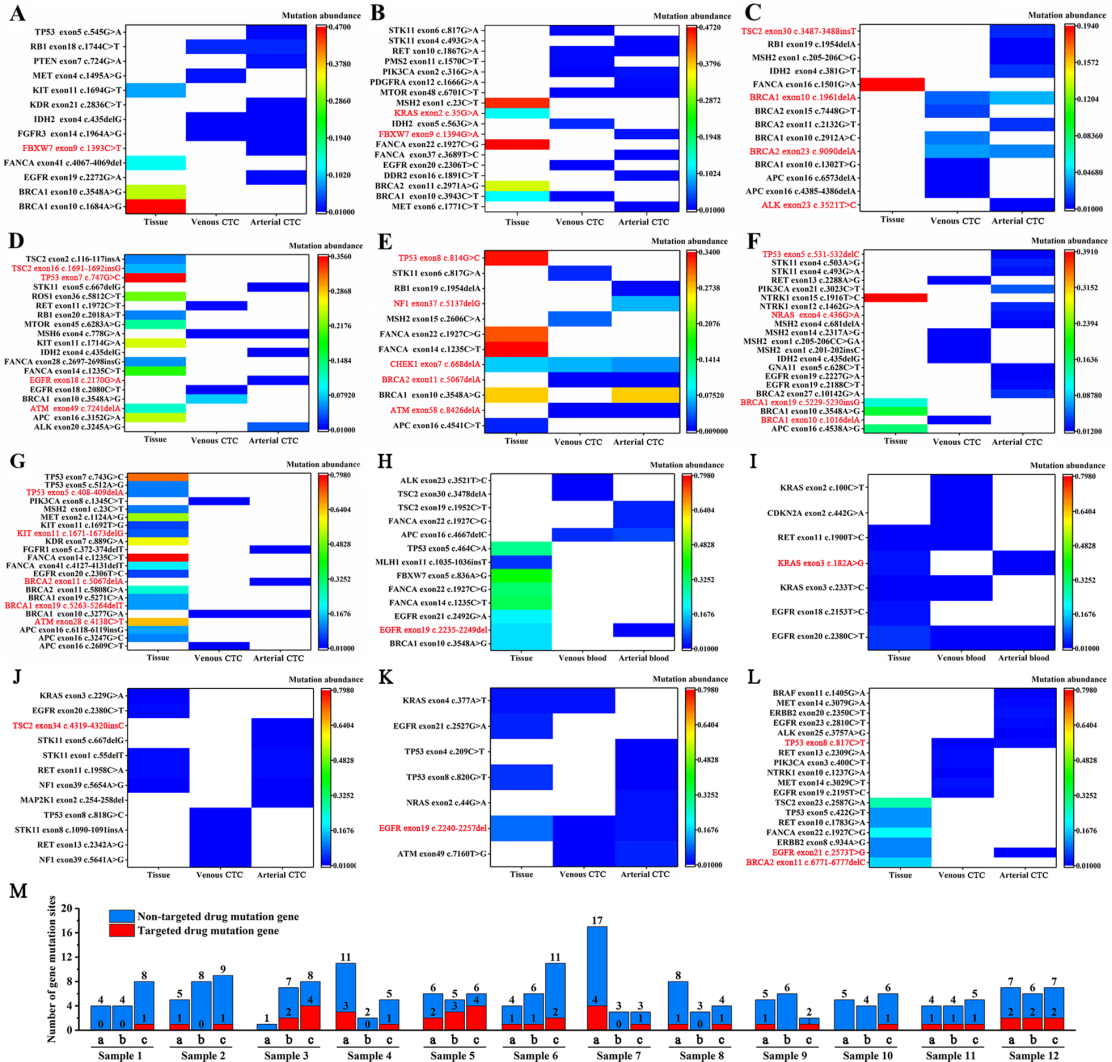
Detection of gene mutations in tissues, arterial CTCs and venous CTCs of patients with different tumors by NGS. **A** Patients with lung cancer; **B** Patients with colon cancer; **C** Patients with liver cancer; **D** Patients with liver cancer; **E** Patients with liver cancer; **F** Patients with pancreatic cancer; **G** Patients with neuroendocrine carcinoma; **H** Patients with Gastric cancer; **I** Patients with renal cancer; **J** Patients with colorectal cancer; **K** Patients with cholangiocarcinoma; **L** Patients with midline cancer; **M** Statistics of the number of genes detected (a tissue; b venous CTCs; c arterial CTCs)

In this study, 12 cases of CTCs positive samples were collected for gene detection, and tissue samples were detected for comparison. As shown in Fig. 8, genes highlighted in red were mutations with clinically targeted drugs. It can be clearly seen that the abundance of gene mutations in tissues was significantly higher than that detected in arterial CTCs and venous CTCs (Fig. 8A–G), which can be attributed to the fact that the number of tumor cells detected in tissues was significantly higher than that detected in arterial blood and venous blood. As shown in Fig. 8A and Additional file 3: Figure S3A, more mutated genes can be detected in arterial CTCs of patients with lung cancer compared with tissues and venous CTCs, and the mutated genes targeted with clinically available drugs (Fbxw7) can only be detected in arterial CTCs. As shown in Fig. 8B and Additional file 3: Figure S3B, arterial CTCs and venous CTCs of patients with colon cancer can detect more mutated genes than tissue, and arterial CTCs can detect the mutated genes targeted with clinically available drugs (Fbxw7), while tissues can also detect the mutated genes targeted with clinically available drugs (KRAS). As can be seen from Fig. 8C and Additional file 3: Figure S3C, arterial CTCs and venous CTCs can detect more mutated genes than tissues, and arterial CTCs can detect the mutated genes targeted with clinically available drugs (TSC2, BRCA1, BRCA2, ALK), while venous CTCs can also detect mutated genes targeted with clinically available drugs (BRCA1, BRCA2); As can be seen from Fig. 8D and Additional file 3: Figure S3D, compared with arterial CTCs and venous CTCs, more mutated genes can be detected in the tissues of patients with liver cancer, and the mutated genes targeted with clinically available drugs (TSC2, TP53, ATM) can be detected in tissues. At the same time, the mutated genes targeted with clinically available drugs (EGFR) can also be detected in arterial CTCs. As can be seen from Fig. 8E and Additional file 3: Figure S3E, the number of mutated genes detected in tissues, arterial CTCs and venous CTCs in patients with liver cancer was basically the same. The mutated genes targeted with clinically available drugs (CHEK1) were detected in tissues, arterial CTCs and venous CTCs at the same time, and the mutated genes targeted with clinically available drugs (BRCA2 and ATM) were detected in arterial CTCs and venous CTCs at the same time. However, the two gene mutations were not detected in tissues, and TP53 gene mutation was detected only in tissues and NF1 gene mutation was detected only in arterial CTCs. As can be seen from Fig. 8F and Additional file 3: Figure S3F, more mutated genes were detected in arterial CTCs than tissues and venous CTCs in patients with pancreatic carcinoma, and the mutated genes targeted with clinically available drugs (TP53, NRAS) were detected in arterial CTCs. At the same time, the mutated genes targeted with clinically available drugs (BRCA1 exon19) were detected in tissues, and the mutated genes targeted with clinically available drugs (BRCA1 exon10) were detected in venous CTCs. As can be seen from Fig. 8G and Additional file 3: Figure S3G, the tissues of patients with neuroendocrine carcinoma can detect more mutated genes than arterial CTCs and venous CTCs, and multiple mutated genes (TP53, KIT, BRCA1, ATM) targeted with clinically available drugs were detected in tissues. At the same time, the mutated genes targeted with clinically available drugs (BRCA2) were detected in arterial CTCs. Figure 8H shows the detection of targeted drug mutation genes and non-targeted drug mutation genes in seven patients. As can be seen from Fig. 8H and Additional file 3: Figure S3H, more mutated genes can be detected in tissues of patients with gastric cancer compared with arterial CTCs and venous CTCs, the mutated genes targeted with clinically available drugs (EGFR exon19) were detected in tissues and arterial CTCs at the same time. As can be seen from Fig. 8I and Additional file 3: Figure S3I, tissue and venous CTCs of patients with renal cancer can detect more mutated genes than arterial CTCs, the mutated genes targeted with clinically available drugs (KRAS exon3) were detected in tissues and arterial CTCs at the same time, the mutated genes (EGFR exon20) were detected in tissues, arterial CTCs and venous CTCs at the same time. Figure 8J and Additional file 3: Figure S3J, the number of mutated genes detected in tissues, arterial CTCs and venous CTCs in patients with colorectal cancer was basically the same, and TSC2 exon34 gene mutation targeted with clinically available drugs was detected only in arterial CTCs. Figure 8K and Additional file 3: Figure S3K, the number of mutated genes detected in tissues, arterial CTCs and venous CTCs in patients with cholangiocarcinoma was basically the same, the mutated genes targeted with clinically available drugs (EGFR exon19) were detected in tissues, arterial CTCs and venous CTCs at the same time. Figure 8K and Additional file 3: Figure S3K, the number of mutated genes detected in tissues, arterial CTCs and venous CTCs in patients with midline carcinoma was basically the same, the mutated genes targeted with clinically available drugs (EGFR exon19) were detected in tissues, arterial CTCs and venous CTCs at the same time.

According to Fig. 8M, the number of arterial CTCs genes detected in samples 1, 2, 3, 6, 10 and 11 were greater than that of tissues and venous CTCs, and the number of clinically significant targeted drug mutation genes detected was higher than that of tissues and venous blood. The number of arterial CTCs and tissue mutation genes detected in sample 5, 10, 11 and 12 was the same, but the number of clinically significant targeted drug mutant genes detected by arterial blood CTCs was still higher than that of tissues. The total number of mutated genes and the number of clinically significant targeted drug mutated genes detected in tissue samples 4, 7 and 8 were higher than those in arterial CTCs and venous CTCs. These results all indicate that there is great heterogeneity among tumor cells of different origins. These results indicate that there is great heterogeneity among tumor cells from disparate sources. At the same time, we found that in samples 1–12, gene mutation targeted with clinically available drugs could be detected in arterial CTCs, which indicates that the arterial CTCs gene test results have more clinical value.

## Discussion

The metastasis process is different from the occurrence and development of cancer, because not all transformed cells will metastasize. Current research shows that some transformed cancer cells, possibly some cancer progenitor cells, will produce metastatic forms of cancer through EMT. This process is very complicated and is regulated by multiple mechanisms. Since metastasis includes EMT and MET, we believe that this should be a reversible phenomenon [17–19]. CTCs is an important marker for clinical prognosis evaluation. CTCs detaches from the primary tumor in the form of single cells or cell clusters (also known as circulating tumor microemboli, CTM) in the blood circulatory system. Most CTCs are in the process of detaching from the primary tumor. EMT occurred in [26]. CTCs, as a potential “seed” that causes tumor metastasis, has attracted extensive attention in recent years [27, 28]. The detection of CTCs depends on the sensitivity of the separation technique. At present, the only approved CTCs detection method is the CellSearch system, which uses EpCAM-coated magnetic beads to enrich CTCs and use anti-CK antibodies for identification, but the positive rate of CTCs detected by this method is low [29–31]. Therefore, we have constructed a new CTCs separation system, and research shows that the capture efficiency of this system is 4.4 ± 1.2 times higher than that of the Cellsearch system [32]. Studies have shown that vimentin has become a potential target for capturing CTCs in patients with tumor [15, 16]. In view of this fact, an accurate and efficient CTCs analysis method based on EpCAM+/Vimentin+ is developed in this study. The results showed that the separation system can efficiently capture CTCs in blood, and can simultaneously capture tumor cells before and after EMT transformation.

In recent years, studies have detected CTCs in five key vascular sites in patients with localized liver cancer. The results show that CTCs in hepatic vein are significantly higher than that in the inferior hepatic vein and portal vein, indicating that the hepatic vein is the for primary tumor cells to enter the circulation [19]. There have also been studies comparing CTCs counts of multiple vascular septa in breast cancer and colorectal cancer [33]. Despite the fact that relevant studies have been carried out on CTCs from disparate sample sources, no CTCs studies based on arterial and venous blood sources have been reported, and the clinical correlation between the distribution pattern of CTCs in arterial and venous blood of tumor patients remains unclear. In this study, the number of CTCs in arterial blood and venous blood was used for the first time to study the relationship between the prognosis of patients, and gene detection was also used for the first time to compare the gene mutation in clinical samples of tissue, arterial CTCs and venous CTCs. The results showed that the number of arterial CTCs is higher than that of venous CTCs, with a statistically significant difference (*p* < 0.05); Epithelium and stroma CTCs are mainly concentrated in arterial blood, and the prognosis of the low positive group of total CTCs in arterial blood and venous blood was superior to that of the high positive group, with a statistical significance (*p* < 0.05). It was shown in the genetic test results that the targeted drug gene mutations in tissues, arterial CTCs and venous CTCs show a complementary trend, indicating that there is heterogeneity among different tumor samples, At the same time, we found that in all patients enrolled, gene mutation information with clinical drug significance could be detected in arterial CTCs, which indicates that the arterial CTCs gene test results have more clinical value. The conclusion of this study can provide a technical reference for efficacy evaluation, prognosis judgment and detection of micrometastasis in patients with cancer. At the same time, thereby it provided a reference basis for gene mutation detection prior to clinical targeted drug therapy. Nevertheless, limited clinical sample size is used for genetic testing in this study. For this reason, countermeasures need to be taken to further increase the sample size verification of consistency of gene mutations in samples from disparate sources, which are conducive to providing more accurate detection means for clinical targeted drug therapy.

## Conclusion

CTCs in blood can be efficiently captured by the CTCs sorting system based on Vi-LMB/Ep-LMB developed in this study, and CTCs detection in arterial blood can be utilized to more accurately determine the disease condition, evaluate the prognosis and predict the postoperative progress. It is further confirmed that tumor samples from disparate sources are heterogeneous, providing a reference basis for gene mutation detection before clinical targeted drug treatment, and the detection of CTCs in arterial blood has more potential clinical application value.

## Materials and methods

### Specimen source

Sixty-eight patients with advanced cancer who received interventional surgery in our hospital from December 2018–April 2021 were collected, including 29 male patients and 39 female patients, aged from 50 to 99 years old, with a median age of 77 years old and an average age of 76.7 years old. During interventional surgery, 7.5 mL of arterial blood and 7.5 ml of venous blood were collected from patients via medical anticoagulant blood collection vessels, and the anticoagulant was EDTA▪K2. The samples were stored at 4 °C to avoid freezing during storage, processing and transportation, and were detected within 72 h. Tissue samples were collected by puncture with each needle larger than 1 mm × 1 mm × 20 mm. After soaking in formalin, the samples were transported at room temperature, and DNA was extracted within 48 h. Meanwhile, 20 healthy volunteers were recruited and 7.5 mL venous blood was taken as negative control. This study was carried out under the approval of The Ethics Committee of Putuo Hospital (PTEC-A-2019-18-1). The patients provided written consent after receiving verbal and written information regarding the study.

### Materials and instruments

Gastric cancer cell line (HS-746T), liver cancer cell line (huh7), lung cancer cell line (A549), and pancreatic cancer cell line (PANC-1) were obtained from the Shanghai Cell Bank of the Chinese Academy of Sciences. Fe_3_O_4_ solution, hexadecyl-quaternized (carboxymethyl) chitosans (HQCMC), CK19-FITC, CD45-PE, DAPI and EpCAM modified lipid magnetic balls (Ep-LMB) [34] were purchased from Huzhou Lieyuan Medical Laboratory Co., Ltd.; A Prussian blue staining kit was purchased from Solarbio; Distearoyl phosphoethanolamine-PEG (DSPE-PEG) was purchased from Avanti (USA); DMEM culture medium, fetal bovine serum and trypsin were purchased from Gibco; EpCAM antibody and Vimentin antibody were purchased from eBioscience. Moreover, 1,2-Dioleylphosphatidylcholine (DOPC), dimethyl octadecyl epoxypropyl ammonium chloride (GHDC), cholesterol, dichloromethane, N-hydroxysuccinimide (NHS), 1-ethyl 3-(3-dimethylammonium propyl) ammonium bicarbonate (EDC) and other commonly used reagents were purchased from Sinopharm (China); A TIANamp Genomic DNA kit was purchased from TIANGEN (BEIJING) BIOTECH; The BI‐90Plus laser particle size analyzer/Zeta potentiometer was purchased from Brooke‐Haiwen, USA; An OLYMPUS B ×61 fluorescence microscope was purchased from Olympus, Japan.

### Preparation of magnetic balls

DSPE-PEG (10 mg), cholesterol (10 mg), DOPC (10 mg), HQCMC (5 mg) were dissolved in 2 mL dichloromethane with full dissolution first. After 1 mL Fe_3_O_4_ solution (36.4 mg/mL) and 0.1 mol/L PBS (pH = 7.4) were added, the mixed solution was ultrasonically oscillated using an ultrasonic instrument with a power of 27% at 25 °C (total time 6 min and rotary evaporation for 30 min), so as to remove dichloromethane to obtain lipid magnetic ball (LMB) aqueous solution. 1 mg GHDC was dissolved in 1 mL isopropanol, and 60 µg Vimentin was dissolved in 1 mL GHDC solution, with the addition of the coupling agents N-hydroxysuccinimide (NHS) and 1-ethyl-3-(3-dimethylaminopropyl) carbodiimide (EDC) respectively. The 1 mL Vimentin-GHDC was dissolved in 1 mL LMB overnight at 4 °C, and then stored at 4 °C for 5 min after swirling. The mixture was taken out and swirled for 5 min every hour for 24 h. In the end, a lipid magnetic ball modified with Vimentin peptides was obtained and designated as Vi-LMB [32, 35, 36].

### Characterization test

After 10 μL of the sample was diluted in 1 mL distilled water, the particle size and potential of the magnetic ball were measured with a BI-90Plus laser particle size analyzer/Zeta potentiometer. Then, the ultraviolet absorption spectrum of the lipid magnetic ball was detected by an ultraviolet spectrophotometer. After 1 mL sample was lyophilized, KBr tablet was used to prepare the sample, and the infrared spectrum was measured by Fourier transform infrared (FT-IR) spectroscopy on a Bio-Rad FTS 6000 spectrometer. After taking 10 μL of the sample and diluting it in 1 mL of distilled water, 50 μL of the sample was applied to the mica sheet and then dried naturally. The morphology of the lipid magnetic ball was measured by an atomic force microscope (AFM). 50 μL of the diluent was dropped on the copper grid, and the morphology of the lipid magnetic ball was observed by a transmission electron microscope (TEM) after drying.

### Cytotoxicity

The cells were cultured in RPMll640 medium containing 10% newborn calf serum at 37 °C and 5% CO_2_ in a constant temperature incubator. The cells were prepared into a single cell suspension with trypsin and seeded into a 96-well plate at a density of 8000 cells per well. When the cells grew 80% confluence, they were cultured in 100 μL of complete culture medium containing different amounts of Ep-LMB and Vi-LMB in DMEM, and blank control plus 100 μL of complete DMEM medium. 20 μL of MTT (5 mg/mL) reagent was added and incubated in a carbon dioxide incubator for 3 h. Finally, 150 μL of dimethyl sulfoxide (DMSO) solution was added to dissolve the crystallized formazan. The results were read and counted by a microplate reader at a wavelength of 490 nm, and three parallel tests were performed for each group.

### Distribution of magnetic balls on cell surface

The single cell suspension of HS-746T, huh7, A549 and PANC-1 was prepared. After counting, 100 cells were added into 7.5 mL PBS solutions to simulate CTCs suspension, and the prepared LMB, Ep-LMB and Vi-LMB magnetic balls were used to capture the cells. After capture, the cells were stained with the Prussian blue dye and observed under a fluorescence microscope. At the same time, the captured cells were smeared on the sample mirror and sprayed with gold after the sample dried, and observed with a scanning electron microscope (SEM).

### Detection of cell capture efficiency

A single cell suspension of A549 cells was prepared. After counting, A549 cells were added into PBS solution at the ratio of 100 cells/7.5 mL, and the suspension was divided into five groups: Ep-LMB group, Vi-LMB group, Ep-LMB + Vi-LMB group, Ep-LMB/Vi-LMB group, and Vi-LMB/Ep-LMB group. Ep-LMB group and Vi-LMB group: 10, 15, 20 and 30 μL Ep-LMB magnetic balls were added to 7.5 mL cell suspension respectively; Ep-LMB + Vi-LMB group: Ep-LMB + Vi-LMB was mixed by volume ratio of 1:1. and 10, 15, 20 and 30 μL Ep-LMB + Vi-LMB mixture were added respectively; Ep-LMB/Vi-LMB group: 5, 7.5, 10 and 15 μL of Ep-LMB were added for capture first, and then 5, 7.5, 10 and 15 μL of Vi-LMB were added for capture respectively; Vi-LMB/Ep-LMB group, 5, 7.5, 10 and 15 μL of Vi-LMB were added respectively for capture, and then 5, 7.5, 10 and 15 μL of Vi LMB were added respectively for capture. Experiments were repeated at least three times in each group. Blood was substituted for PBS solution to conduct CTCs capture experiment in simulated blood, and HS-746 T, huh7 and PANC-1 cells were gradient captured in PBS and simulated blood to investigate the stability of magnetic bead capture efficiency (10, 50, 100, 200, 500 and 1000 cells). Finally, Ep-LMB and Vi-LMB with different antibody content are gradient capture of A549 cells (antibody content: 0 μg, 10 μg, 20 μg, 30 μg, 40 μg, 50 μg, 60 μg, 70 μg, 80 μg, 90 μg and 100 μg), investigate the capture efficiency of magnetic beads with different antibody content on cells.

### Exploration of cell capture time

A total of 1 × 10^4^ A549 cells were inoculated in a petri dish, and 1 mL of cell culture medium was added. The cells were cultured in a thermostatic incubator at 37 °C with 5% CO_2_ for 24 h. After replacing the culture medium, 20 μL Ep-LMB-FITC or Vi-LMB-FITC, 100 μL DAPI and 100 μL Dil were added, and the petri dish was fixed on and photographed with a fluorescence microscope at 0 min, 5 min, 10 min, 15 min and 20 min respectively.

### Isolation and identification of CTCs in clinical blood samples

Clinical samples of 68 patients were collected, and 7.5 mL arterial blood and 7.5 mL venous blood were taken respectively. First, 10 μL of Ep-LMB was added and incubated at room temperature for 15 min, and was mixed once every 5 min. After the incubation, the centrifuge tube was inserted into the magnetic separator to adsorb for 10 min, after which the supernatant was discarded. Then, 10 μL of Vi-LMB was added and incubated at room temperature for 15 min, and was mixed once every 5 min. After the incubation, the centrifuge tube was inserted into the magnetic separator to adsorb for 10 min, after which the supernatant was discarded. 1 mL PBS was used to perform a magnetic separation wash on the two captured CTCs samples twice to obtain Arterial CTCs and Venous CTCs, respectively. Subsequently, 20 μL of FITC-labeled CK19 monoclonal antibody (CK19-FITC), 20 μL of DAPI staining solution and 20 μL of PE-labeled CD45 antibody (CD45-PE) were added and mixed uniformly and stained for 15 min without light. After staining, 1 mL of ddH_2_O was added for washing twice, and 20 μL of ddH_2_O was added to the centrifuge tube or mixing. The obtained mixture was smeared evenly on the polylysine treated anti-slip slide, and the droplets were observed and counted under a fluorescence microscope after being naturally dried.

### Gene detection assay

The fresh tissue, 7.5 mL of arterial blood and 7.5 mL of venous blood were collected from 7 patients randomly. Artistic CTCs were captured by Ep-LMB and Vi-LMB, respectively, and venous CTCs were captured by Ep-LMB and Vi-LMB, respectively. The CTCs in arterial blood and venous blood were combined, and the total DNA in Arterial CTCs and Venous CTCs was extracted by the TIANamp Genomic DNA Kit (total tissue DNA > 200 ng, total CTC-DNA > 20 ng). Target area capture technology combined with “Next-generation” sequencing (NGS) technology was utilized in this detection to detect the samples, covering 210 related genes including single nucleotide variations, short segment insertion or deletion variation, gene copy number variation and gene rearrangement within the coverage of the probe in the target gene capture region, with about 5,700 mutant gene loci.

### Statistical analysis

All statistical analyses in this study were performed using SPSS 21.0 software. Survival curve was made according to survival time and survival rate, and the relationship between the number of arterial CTCs and venous CTCs and survival time was investigated. The Kaplan–Meier method was used for univariate survival analysis, and the log rank test was used for survival rate comparison. P < 0.05 was considered statistically significant (*), and P < 0.01 was considered extremely significant (**).

## Supplementary Information

**Additional file 1: Figure S1.** Effects of different concentrations of Ep-LMB and Vi-LMB on the cell viability of different cancer cells. A. Effect of Ep-LMB on cell activity of different cancer cells; B. Effect of Vi-LMB on cell activity of different cancer cells.

**Additional file 2: Figure S2.** Detection of capture efficiency of Ep-LMB and Vi-LMB for A549, HS-746T, huh7 and PANC-1 cells in different systems. A. Detection of capture efficiency of A549 cells by different amounts of Ep-LMB and Vi-LMB in PBS; B. Detection of capture efficiency of A549 cells by Ep-LMB and Vi-LMB magnetic beads with different usage in simulated blood; C. Stability detection of capture efficiency of A549, HS-746T, huh7 and PANC-1 cells by Ep-LMB and Vi-LMB in PBS; D. Stability detection of capture efficiency of A549, HS-746T, huh7 and PANC-1 cells by Ep-LMB and Vi-LMB in simulated blood; E. The capture efficiency of magnetic balls with different antibody content on A549 cells.

**Additional file 3: Figure S3.** Detection of number of genes in tissues, arterial CTCs and venous CTCs of patients with different tumors by NGS. A. Patients with lung cancer; B. Patients with colon cancer; C. Patients with liver cancer; D. Patients with liver cancer; E. Patients with liver cancer; F. Patients with pancreatic cancer; G. Patients with neuroendocrine carcinoma; H. Patients with Gastric cancer; I. Patients with renal cancer; J. Patients with colorectal cancer; K. Patients with cholangiocarcinoma; L. Patients with midline cancer.

## Data Availability

All data generated or analyzed during this study are included in this published article and its supplementary information files.
