# Supplementary figures and images for: Clinical verification of vimentin/EpCAM immunolipid magnetic sorting system in monitoring CTCs in arterial and venous blood of advanced tumor
Source: J Nanobiotechnology. 2021 Jun 16;19:185. doi: 10.1186/s12951-021-00929-x (PMC8207779; doi:10.1186/s12951-021-00929-x)

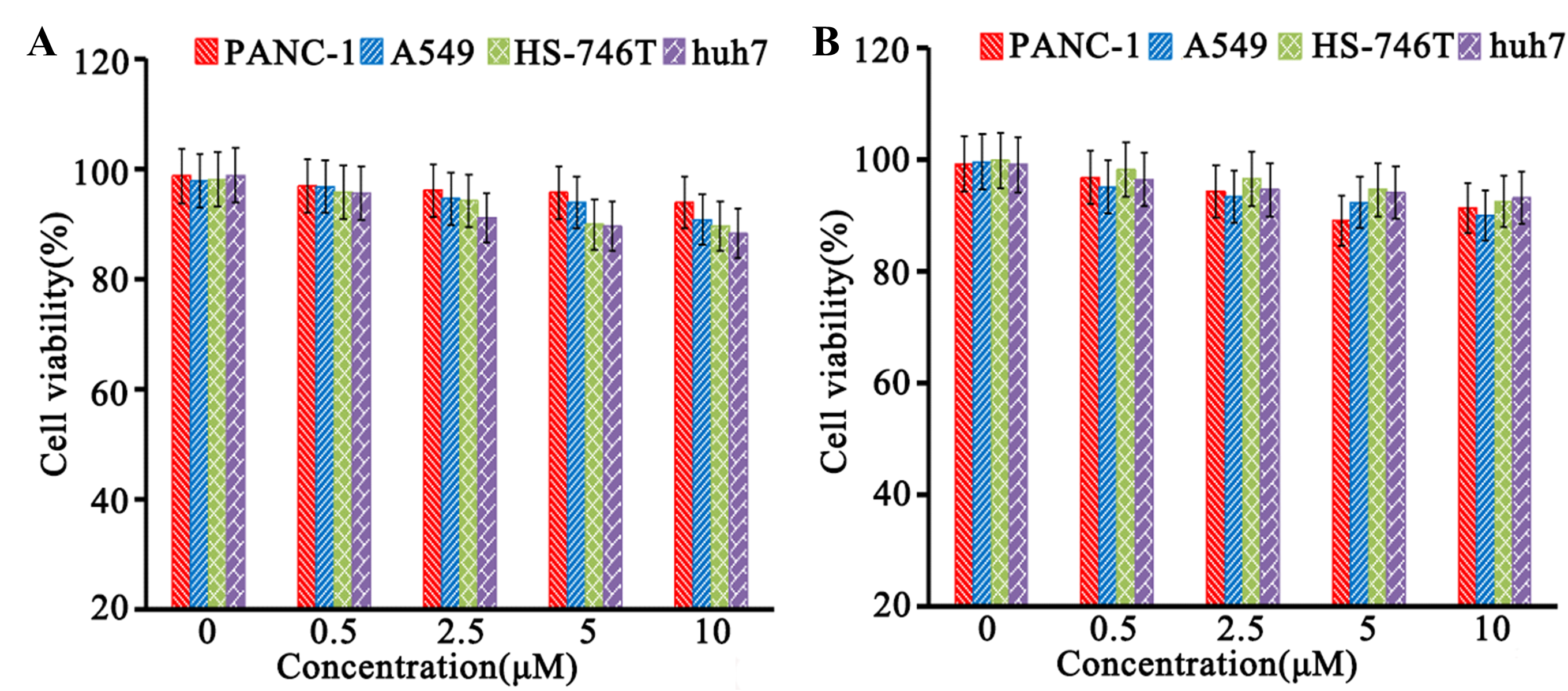

Supplement: Supplementary file 1 — Additional file 1: Figure S1. Effects of different concentrations of Ep-LMB and Vi-LMB on the cell viability of different cancer cells. A. Effect of Ep-LMB on cell activity of different cancer cells; B. Effect of Vi-LMB on cell activity of different cancer cells. [file 12951_2021_929_MOESM1_ESM.tif]

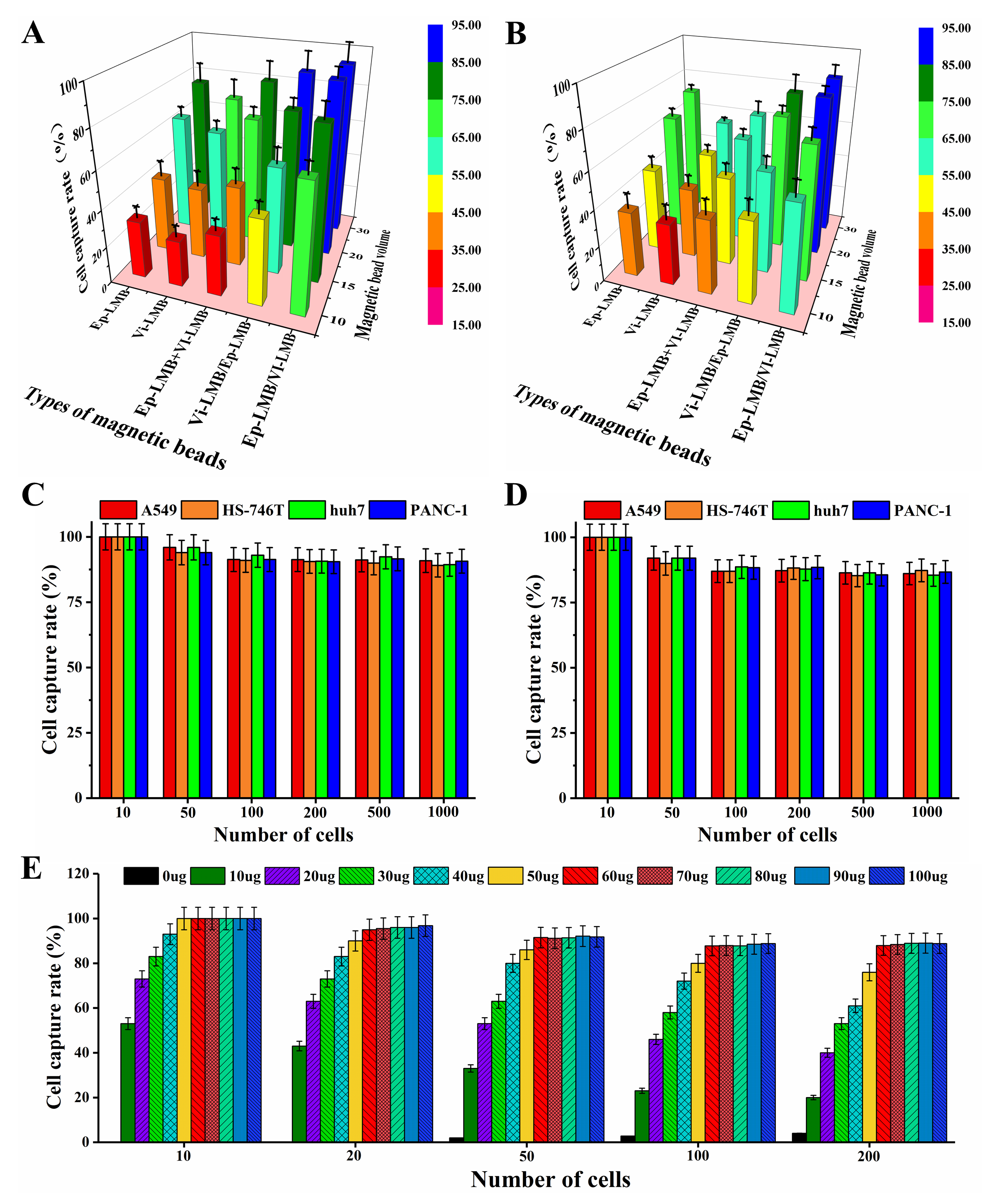

Supplement: Supplementary file 2 — Additional file 2: Figure S2. Detection of capture efficiency of Ep-LMB and Vi-LMB for A549, HS-746T, huh7 and PANC-1 cells in different systems. A. Detection of capture efficiency of A549 cells by different amounts of Ep-LMB and Vi-LMB in PBS; B. Detection of capture efficiency of A549 cells by Ep-LMB and Vi-LMB magnetic beads with different usage in simulated blood; C. Stability detection of capture efficiency of A549, HS-746T, huh7 and PANC-1 cells by Ep-LMB and Vi-LMB in PBS; D. Stability detection of capture efficiency of A549, HS-746T, huh7 and PANC-1 cells by Ep-LMB and Vi-LMB in simulated blood; E. The capture efficiency of magnetic balls with different antibody content on A549 cells. [file 12951_2021_929_MOESM2_ESM.tif]

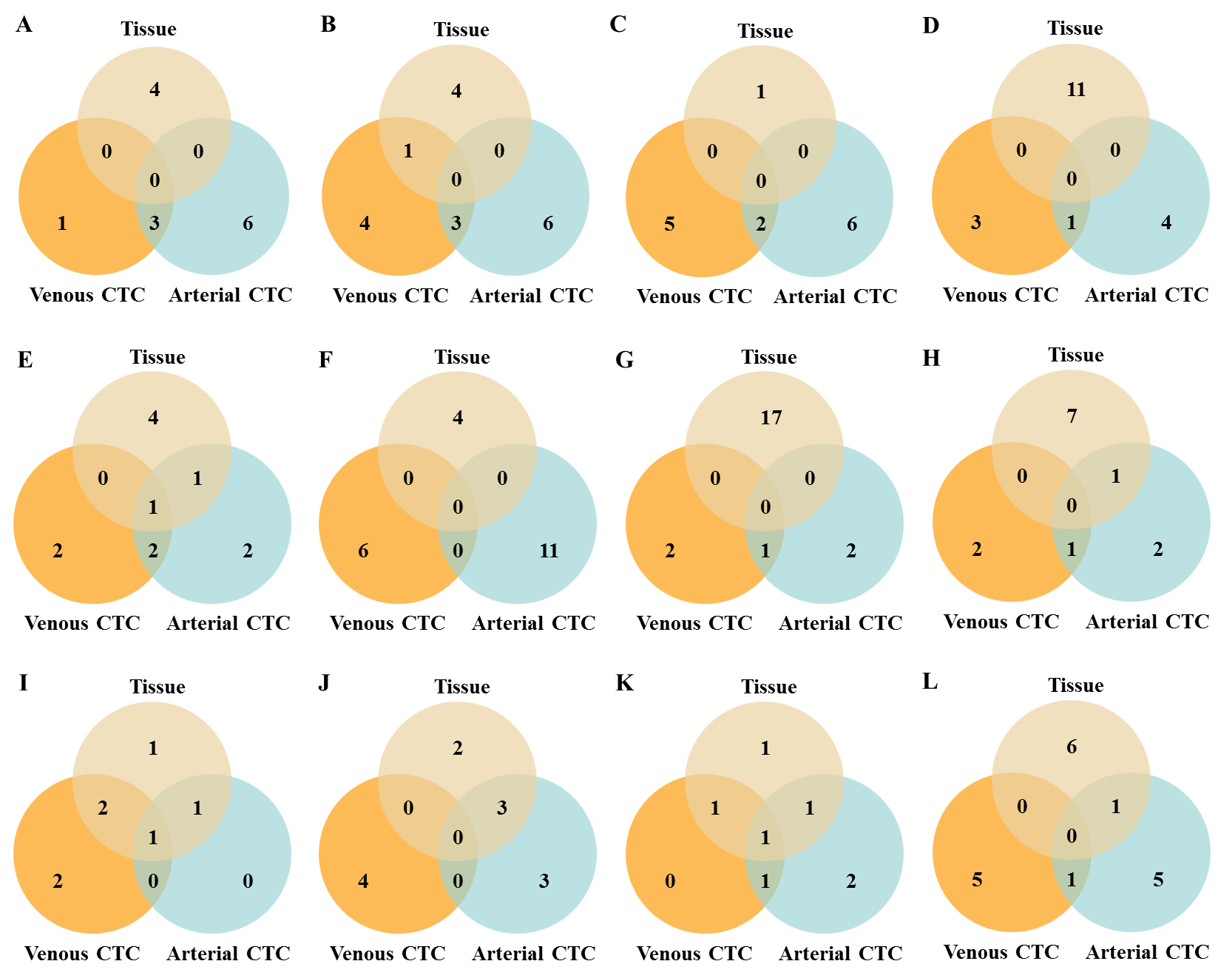

Supplement: Supplementary file 3 — Additional file 3: Figure S3. Detection of number of genes in tissues, arterial CTCs and venous CTCs of patients with different tumors by NGS. A. Patients with lung cancer; B. Patients with colon cancer; C. Patients with liver cancer; D. Patients with liver cancer; E. Patients with liver cancer; F. Patients with pancreatic cancer; G. Patients with neuroendocrine carcinoma; H. Patients with Gastric cancer; I. Patients with renal cancer; J. Patients with colorectal cancer; K. Patients with cholangiocarcinoma; L. Patients with midline cancer. [file 12951_2021_929_MOESM3_ESM.tif]
